# The neurology of chronic nodding syndrome

**DOI:** 10.1093/braincomms/fcac126

**Published:** 2022-06-06

**Authors:** Sam Olum, Charlotte Hardy, James Obol, Neil Scolding

**Affiliations:** Faculty of Medicine, Gulu University, c/o Guest House, Gulu, Uganda; Faculty of Medicine, Gulu University, c/o Guest House, Gulu, Uganda; Emergency Medicine Department, Royal United Hospital, Bath, UK; Institute of Clinical Neurosciences, University of Bristol, Bristol, UK; Faculty of Medicine, Gulu University, c/o Guest House, Gulu, Uganda; Faculty of Medicine, Gulu University, c/o Guest House, Gulu, Uganda; Institute of Clinical Neurosciences, University of Bristol, Bristol, UK

**Keywords:** nodding syndrome, sub-Saharan Africa

## Abstract

Nodding syndrome is an uncommon disorder of childhood onset and unknown cause, presenting with nodding seizures, and which appears to occur exclusively in clusters in sub-Saharan Africa. An endemic pattern of disease was initially described in Tanzania and in Liberia; epidemic occurrences were later reported in South Sudan and northern Uganda. Not the least significant of the many questions remaining about nodding syndrome concerns the common presence or otherwise of neurological features other than seizures—clearly relevant to the core issue of whether this is a focal, primary epileptic disease, or a multi-system CNS disorder, with, in turn implications for its aetiology. We had the opportunity to interview and clinically to examine 57 affected individuals in rural northern Uganda some 10 years after onset. In this observational cross-sectional study, nodding onset was invariably between the ages of 5 and 14, presenting with food-triggered nodding attacks in over 75% of cases; 86% went on to develop other seizure types. In 53 of 57 nodding syndrome individuals (93%), there was a definite history of the child and his or her family having resided in or been fed from an internally displaced person camp for some time prior to the onset of nodding. A half of nodding syndrome sufferers (28/57) had focal neurological abnormalities—mainly pyramidal signs (92%), often asymmetric, some with extrapyramidal abnormalities. Many individuals (28/57) were severely functionally disabled, ranging from ‘sometimes can dig’ to ‘can do nothing at home’ or ‘cannot even feed herself’. Such sufferers tended more frequently to have significant burns, and clear cognitive impairment. We conclude that nodding syndrome is a unique multisystem CNS disorder of childhood onset and then slow progression over several years often followed by spontaneous stabilisation, consistent with an underlying self-limiting neurodegenerative process. We discuss the possibility that this might be triggered by food-related mycotoxins, within a fixed window of CNS vulnerability during childhood.

## Teaser

Nodding syndrome is a poorly understood, predominantly epileptic disorder identified almost exclusively in rural East Africa. Focal neurological signs have not been studied. Scolding *et al.* examined 57 individuals with chronic nodding syndrome in rural settings in northern Uganda and found pyramidal and/or extrapyramidal signs in almost half.

## Graphical Abstract

**Graphical Abstract:**
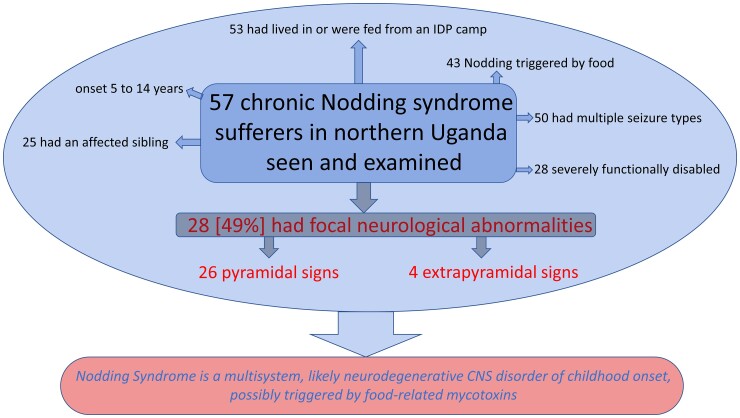

## Introduction

Nodding syndrome (NS) is a serious childhood-onset epileptic disorder of unknown cause, occurring in clusters principally in sub-Saharan Africa. It was first described in the early 1960s in Tanzania^1^ and has subsequently been observed and studied mainly in South Sudan, Tanzania and northern Uganda.^2–6^ NS is characterized (and defined) by episodic repetitive head nodding, 5–20 nods per minute, resulting from momentary but repeated loss of neck extensor tone, for which an epileptic origin was proposed^1^ and then ultimately confirmed.^5^ Occasionally, an associated loss of tone in the upper trunk and shoulders occurs. Notably, nodding episodes are commonly triggered by presentation with food, or by cold.^1–3,5^ In some cases, nodding is associated with impaired awareness; other affected individuals continue eating and interacting between nods during episodes.^3^ Other forms of epileptic seizure usually follow, including focal impaired awareness seizures, tonic-clonic, and atypical absence seizures. Seizure-related death is not infrequently seen as a consequence of burns from domestic fires, or drowning.

It has been clear since the outset that NS is not characterized solely by seizures.^1,7^ Failure of cognitive development and physical growth retardation have been consistently described as associated late features^2,3^—although it is unclear whether these are primary components of the disorder or secondary consequences of nutritional, educational, and other social factors. Similarly, various psychological and psychiatric problems, including mood changes, sleep disturbance, major depression, aggressive outbursts, and episodes of wandering, are also seen,^8^ but these too may represent reactive secondary consequences or be partially explained by seizure events.

Chronic and persistent or progressive neurological abnormalities are mentioned in some reports but have not received primary attention in published descriptions, notwithstanding Jilek-Aall’s original (1965) report describing ‘gait disturbance and clumsy movements’ (which she attributed to vitamin deficiencies). Pyramidal, extrapyramidal, and cerebellar signs are also alluded to in Liberian NS cases,^4,7^ although latterly, neurological signs have come to be considered very uncommon.^2,3,9^

The natural history also remains unclear. Most sufferers progress in the initial months from nodding attacks to other seizure types, some with additional (though possibly secondary) cognitive, psychological, and/or physical problems.^9^ However, anecdotal reports depict in some cases a rapidly progressive course from the onset of nodding, with rapidly increasing neurological and cognitive disabilities and then progressive deterioration of consciousness and death.^9–11^ But one of the few peer-reviewed studies with follow-up (12 Ugandan patients over 8 months) reported no general neurological deterioration, albeit some worsening in seizures (frequency or type) in six patients, no improvement in any, and no deaths.^3^ No studies report any instance of spontaneous recovery.

We undertook to study the neurological features of chronic NS by direct clinical examination of affected individuals in northern Uganda. In a series of community-based visits to rural settlements in the Pader and Kitgum area, almost 60 such subjects were identified; aspects of the history were explored with carers, and each affected individual was examined neurologically. Here, we delineate these neurological features, provide some comment on the natural history and, based on the stories we elicited, speculate on the aetiology of this unusual but potentially informative disease.

## Methods

### Visits

This is an observational cross-sectional study based on the findings of four community-based visits undertaken over an 18-month period to rural settlements in the Pader and Kitgum area. Three were organized with the involvement and assistance of the Diocese of Kitgum, diocesan healthworkers having identified subjects (and their carers) within various local communities. For the fourth, in the Pader area, local community-based health workers similarly identified subjects and their carers. Every visit was undertaken by at least one neurologist (N.S.), who examined every affected individual; C.S., S.O., and J.O. also attended visits, examined patients, and recorded aspects of the history. Translation was by S.O., J.O. and local healthworkers.

Neurological examination was slightly limited due to lack of privacy (encounters with subjects and carers invariably took place in an outdoor setting) and also by the language barrier. Ophthalmoscopy was not undertaken.

### Diagnosis

Proposed case definitions for NS (Table 1) were developed at a World Health Organization (WHO)-convened consensus meeting in Kampala in 2012 and follow-up meeting in 2015 in Gulu, Northern Uganda^8,12^; these were applied as possible in the current study.

**Table 1:** Proposed case definitions for nodding syndrome^1^

Suspected case:
(Used at the community level, primarily by marginally trained health teams when asking the mother/caretaker).
• Reported head nodding in a previously normal person. Head nodding is defined as repetitive, involuntary drops of the head to the chest on two or more occasions.
Probable case: Suspect case of head nodding with:
• Both of the following major criteria:
– Age at onset of nodding between 3 and 18 years old
– Frequency of nodding 5–20 per minute
• Plus at least one of the following minor criteria:
– Other neurological abnormalities (cognitive decline, school dropout due to cognitive/behavioural problems, other seizures or neurological abnormalities)
– Clustering in space or time with similar cases
– Triggered by food and/or cold weather
– Stunting or wasting
– Delayed sexual or physical development
– Psychiatric symptoms
Confirmed case: Is a probable case
• Plus a documented nodding episode that is:
– Observed by trained healthcare worker, or
– Videotaped, or
– EEG/EMG.

### Ethics

Ethics permission was granted by Gulu University Research Ethics Committee (GUREC-2021-92).

## Results

### Diagnosis and basic demographics

A total of 67 individuals were seen and examined neurologically during the four visits (Supplementary Table). On the basis of the history—mostly from carers, partly from affected individuals—and applying the WHO diagnostic criteria (Table 1), 10 of these appeared highly unlikely to meet the criteria for *Suspected NS* and were excluded from further analysis. (In none of these 10 cases did carers or individuals report any incidence of clear nodding episodes. Some appeared quite clearly to have other forms of generalized epilepsy, others may have represented cases of non-epileptic seizures.)

There were therefore 57 individuals with *Suspected NS*. Of these, once seen and assessed, all were judged to be *Probable* NS (although no more than a crude estimate of the nodding frequency was possible—‘frequency of nodding 5 to 20 per minute’ being one of the two mandatory major criteria; Table 1). No cases could be classified as *Confirmed* NS since no episodes of nodding were witnessed (although several individuals had witnessed tonic clonic seizures whilst waiting to be seen), and it was not possible to ascertain whether or not nodding in individual cases had been reliably observed by trained healthcare workers; neither were ‘videotaped’ episodes available, nor were EEG/EMG recordings feasible in these outdoor rural settings.

Seven of the 57 individuals had nodding attacks but no other seizure types; four of these had no clinical signs and no apparent disability. [They were classified as ‘probable’ rather than ‘suspected’ either because nodding was induced by food or cold, or because of ‘clustering in space or time with similar cases’ (or both).]

Of the 57 individuals with probable NS, 32 were males and 25 females. The age when seen ranged from 14 to 26 years (mean 18.9 years). The age at onset of symptoms ranged from 5 to 14 years (mean 8.4 years; median 6 years). The average duration of the illness in these affected individuals was therefore over ten years (10.5y).

### Background history

Our study was intended primarily to explore neurological features, but we were able to ascertain some information relating to aetiological hypotheses for NS. In all but four cases (53/57, 93%) there was a definite history of the child and his or her family having resided in an internally displaced person (IDP) camp for some time prior to the onset of nodding or, in some cases, having lived nearby but obtaining all their food from the IDP.

As others have noted, affected sibships are common—25/57 (43.8%) had an affected sibling, 13/57 (22.8%) definitely did not. In the remaining 19 cases, this information was not collected.

### Nodding and seizure episodes

Nodding was explicitly triggered by food in 43 cases (75.4%); cold as a trigger was described in 8 cases (14%). A typical description (a male sufferer, onset of nodding aged 6y) was that ‘when he started eating, his neck twisted to look away from food, then he started nodding. Then he would say “there is nothing wrong,” then eat, and then go and play’.

In the 50 individuals who also had other seizure types, the description of the other seizures was often extremely clear. For example (Case 30) ‘he rolls his eyes, then falls and passes water. He is stiff, then has jerking of all four limbs. There is drooling of saliva. Then he sleeps’. In another case (Case 1) ‘sometime there is no warning or sometimes he may be confused for a minute or two; there is no trigger; he makes an expirational noise, then loses consciousness, with urine incontinence. He recovers around 1 h later but often sleeps’. Or again (Case 4) ‘He suddenly falls but gets straight back up again’, with no jerking and no suggestion of loss of consciousness. Many clearly had more than one seizure type. In other cases, however, language difficulties, often with the complicating factor of a non-primary carer, made it extremely difficult to establish the semiology of the additional seizures. Often the descriptors were no more than a single word—‘jerking’ or ‘falling’ attacks. Our classification was necessarily therefore rather subjective. We labelled attacks as generalized tonic-clonic convulsions if there were clear features (as in the quoted descriptions above) or if descriptors such as ‘jerking’ and ‘falling’ were *both* used. If only one of these terms was used, or if the information was otherwise insufficient to make a judgement, we considered the episodes unspecifiable—this was the case in seven individuals.

Forty (70.1%) of the total 57 cases had generalized tonic-clonic convulsions. Four (7%) had absence attacks; two (3.5%) appeared to have atonic attacks.

An at least partial drug history was available in 49 individuals; 47 of these were on some form of treatment, but only in a minority of cases was it possible to identify which drug—carbamazepine in 10 cases, sodium valproate in 9.

### General condition, functional abilities and disabilities

We obtained a certain amount of information concerning affected individuals’ level of function and activities of daily living.

Around a fifth had obvious signs of significant previous burns, almost invariably from fires (used for cooking). Two had obvious signs of fetters or ties at the waist or ankle, not uncommonly used as a means of preventing the affected individual from wandering into the bush, or towards and into cooking fires.

A characteristic physiognomy and habitus was apparent. Affected individuals very frequently said little or nothing. Four were described as ‘almost mute’ or ‘cannot talk at all’, others were said to ‘talk a little’. Many constantly looked downwards; most showed very little expression or animation, and drooling was not uncommon. Sufferers were almost invariably very reluctant to engage. It was difficult to establish whether these features reflected mood disturbance with depression, psychomotor retardation, possible extrapyramidal involvement, or perhaps more simply a behavioural reaction—being rather overwhelmed by the highly unfamiliar circumstances of our visit and the clinical examination.

Some individuals (almost a third, 17/57) were said to function well and to be able to fetch water, dig, wash dishes, grind grain, or to play football, for example. At least half (28/57) were described as severely functionally disabled, ranging from ‘sometimes can dig’ to ‘can do nothing at home’ or ‘cannot even feed herself’. Such individuals tended more frequently to have significant burns, neurological signs and clear cognitive impairment (see below). There was no suggestion, from direct enquiry, of any continuing progression of functional disabilities.

Cognition was not formally assessed (see Discussion). Many were described as having been ‘slow in class’ at school, commonly repeating years sometimes several times. Many had dropped out of school altogether at an early stage.

#### Neurological findings

All 67 individuals (including the 10 who did not meet NS diagnostic criteria) were examined. No obvious cranial nerve deficits were found, though there were several significant omissions to a full examination (for example, fundoscopy not done, smell not tested, hearing difficult to assess, swallow not tested). In the limbs, wasting was difficult to assess, as the great majority of individuals was very thin if not emaciated.

Amongst those meeting ‘probable’ NS diagnostic criteria, focal neurological abnormalities (excluding cognitive change) were found in 28/57 (49.1%). The commonest abnormality was an increase in tone, apparent in 26 of 57 NS individuals (45.6%). In most cases this was pyramidal in character (24/26, 92.3%); in four individuals (4/26, 15.4%), the hypertonia was clearly extrapyramidal (two individuals had spastic signs in the lower limbs and extrapyramidal hypertonia in one or both upper limbs). In one of these four, there was additional dystonic posturing of one arm.

In those with pyramidal hypertonia, other pyramidal signs were often but not invariably present—hyper-reflexia in 15/26 (57.7%), reflex spread, positive Hoffman’s sign, clonus, and/or extensor plantar responses in only a few. Impairment of muscle power in all patients was no more than mild—strength very rarely less than Medical Research Council Grade 4.

In 7 of 28 individuals with increased tone (25%), just one limb was affected; in 8 (28.6%) two limbs, 3 (10.7%) three limbs, and 11 (39.3%) individuals had increased tone in all four limbs (all with spasticity). In 18/28 individuals (64.3%), one or both upper limbs were affected; in 23 (82.1%) one or both lower limbs.

No obvious sensory signs were seen, though this was again a difficult part of the examination; joint position sense testing proved not easily translatable, while tuning fork vibration testing did not prove feasible and was abandoned.

## Discussion

An enormous amount has been learned concerning NS since its original description over 55 years ago^1^—but much also remains unknown, and one key question is whether neurological abnormalities are present in longstanding disease, or not. This has clear implications for establishing whether NS is exclusively a primary epileptic disorder, or whether it is a multisystem neurological disease of which epilepsy is a key feature—which distinction in turn carries fundamental implications for understanding the nature and aetiology of the disorder.

Certainly, the presence of neurological abnormalities was suggested in the earlier reports,^1,7^ but later studies indicated that neurological signs were very uncommon.^2,3,9^ Arguably the first report definitively establishing nodding as an epileptic phenomenon suggested that neurological signs (largely unspecified) were found only in 2 or 3 of 62 cases.^5^ Perhaps unsurprisingly therefore, neurological features are given rather little prominence in the accepted WHO diagnostic criteria.^12^

We believe our findings help answer this question definitively in the affirmative: in our study, very nearly half (49%) of the chronic NS patients we examined exhibited focal neurological abnormalities—excluding cognitive change. [We elected not to include consideration of cognitive ability in this assessment principally because while cognitive impairment undoubtedly could reflect neurological damage from (as yet poorly understood) Nodding-related disease processes, it would be extremely difficult without very detailed assessment and preferably longitudinal monitoring to know whether cognitive abilities were impaired as a primary consequence of disease involvement or was instead a secondary phenomenon related to school absence, seizure frequency, social stigmatization, and other consequences of poorly controlled seizures.]

Of those with neurological abnormalities, the great majority had pyramidal signs in one or more limbs, often asymmetrical. The principal manifestation was spastic hypertonia, though other pyramidal signs often but not invariably present—hyper-reflexia in most, with reflex spread, a positive Hoffman’s sign, and clonus rather less common. Weakness was never more than mild—possibly helping to explain why many reports may have overlooked neurological abnormalities. Interestingly, extensor plantar responses were found in only two individuals: in the remainder the plantar reflex was either absent or downgoing. In fact, however, previous authors over many years have commented on the ‘relatively absence’ of the plantar reflex in Africans,^13,14^ though so far this is unexplained. (The British neurologist JD Spillane, examining cases in Ibadan at the invitation of Prof. Ben Osuntoken, is said to have commented ‘The great Babinski would never have discovered his famous sign if he had lived and practised neurology in Africa’.^13^)

A significant minority (some 15%), however, showed clinical evidence of extrapyramidal dysfunction either alone or in addition to pyramidal signs. Others have reported the presence of extrapyramidal signs, particularly Spencer *et al.*^15^ who fascinatingly re-interrogated Jilek-Aall’s original case notes from Mahenge Clinic (Tanzania), records made more than four decades earlier, between 1960 and 1971. These authors suggest that Jilek-Aall had recorded ‘Parkinsonian’ features in ‘one third’ of patients, though this was partly based on the presence of hypersalivation alone, or a ‘mask-like’ face—the latter sign we found particularly difficult to interpret since significant psychomotor retardation was so often a feature. We did, however, find in some individuals a plastic, rigid hypertonia of an extrapyramidal nature, and rarely also dystonic posturing. We observe no instance of tremor (of any nature).

We found no clinical evidence of peripheral nervous system involvement, and no definite involvement of the cerebellar or oculomotor systems (though modest cerebellar disease was difficult to exclude with absolute confidence in those with significant pyramidal abnormalities).

NS therefore appears to be a multi-system CNS disease, with epilepsy as a particularly prominent feature but with variable additional involvement of the pyramidal and extrapyramidal systems—and, with the provisos mentioned above, very likely also with involvement of other cerebral systems resulting in many individuals in significant cognitive impairment.

Some of the discrepancies in the literature between different studies concerning the presence or otherwise of neurological abnormalities might well reflect the stage at which individuals were examined. The average duration of the illness in individuals we examined was over 10 years; sufferers at onset appear unlikely to exhibit such signs.

These findings have implications for the natural history of the disease: the development of neurological signs over a likely time course of several years, combined with the evolution over the first few years of epilepsy to include seizure types other than ‘just’ nodding attacks, implies that NS is both a progressive and a multisystem neurological disorder, the relatively slow time course (deterioration taking place over months to years) more consistent with a neurodegenerative process than with inflammation or infection. That said, it would be an unusual neurodegenerative process whose progress spontaneously halted after a few years, though this appears to be the case in NS: the great majority of individuals we saw appeared to be stable, findings entirely consistent with one of the few studies with follow-up (12 Ugandan patients over 8 months), which reported no general neurological deterioration, some worsening in seizures (frequency or type) in six patients, but no improvement in any, and no deaths.^4^

This unusual time course, with the multisystem nature of NS, has significant implications for understanding the aetiology of the disorder. None of the many hypothesized causes has gained unanimous acceptance: the two main current proposals are infection (or infection-related), and toxins—neither escaping significant critique.^6^ A third, based on the first significant neuropathological autopsy study in NS, proposed that the disorder was a novel tauopathy^16^ but later studies differed, failing to disclose any tau-positive neuronal neurofibrillary tangles or pre-tangles.^17–19^

Onchocerciasis (river blindness) has been repeatedly invoked as a cause: many of the geographical ‘pockets’ of the disease have a high onchocerciasis prevalence,^15^ and case-control studies reported a positive link with onchocercal infection.^20,21^ However, spinal fluid PCR and other CSF tests for O. volvulus in various studies have been consistently negative in all patients.^5,20–22^ In addition, many areas in Africa and elsewhere have a high onchocerciasis prevalence without the occurrence of NS. The recent alternative onchocerciasis-based hypothesis, that the organism triggers auto-immune epilepsy by generating cross-reactive anti-neuronal leiomodin-1 antibodies^23^ again does not accommodate the global incidence of ochocerciasis (in stark contrast to NS). Also, almost half of NS patients have no leiomodin antibodies, while a third of normal controls do have antibodies.

Food-related toxins represent the second main hypothetical cause. An association with the consumption of World Food Programme emergency food, particularly maize which had become mouldy, was reported.^24^ Mouldy food in or from IDP camps was very frequently mentioned in our cohort—93% of whom had resided in or obtained their food from an IDP. In a later study, levels of aflatoxin and ochratoxin in maize were no different to those in any of the grain types tested, and there was no correlation between the total concentration of any of the various types of mycotoxin with the presence of children with NS in households,^25^ although testing was not done at the onset of the disease. It should also be borne in mind that mycotoxins are widespread in core food products throughout Africa, including maize, spices and groundnuts,^26^ again contrasting with the distribution of NS and also militating against their involvement. The time course of the illness would be unlike that of conventional toxins—or, for that matter, infectious processes.

Any theory concerning causation must also accommodate the unusual apparent age-restriction of onset—it is clear from multiple studies that NS invariably begins between the ages of 5 and 1^2–4,6^ (5 and 14 years in the current study). Whilst it is not impossible that an infectious process might have consequences particular to the age of the infected individual, such age-related childhood susceptibility is arguably rather more suggestive of an environmental toxin. Various agents (for example, lead, and certain anaesthetic agents) exhibit neurotoxic effects that are highly dependent on the stage of CNS development and maturation—both pre-natal and post-natally through childhood.^27,28^ The strikingly frequent mention of mouldy food in IDP camps—as in much of the previously published literature, lends no little support to the possibility of a food-related neurotoxin, perhaps a mycotoxin—to which a maturing brain is exclusively susceptible (perhaps, rather speculatively, on a background of previous sub-optimal nutritional status). Such exposure would be consistent with the two epidemic-like occurrences of NS in Northern Uganda and South Sudan, while the more endemic occurrence of NS in Tanzania and Liberia, as Spencer has pointed out, might still have a common origin—‘the NS-susceptible Pogoro people of Tanzania show parallels with the NS-susceptible [South Sudan] population given their probable common …dependence on [foods] subject to spoilage and fungal contamination’.^24^

Understanding rare disease not uncommonly provides major insights into common disorders, perhaps particularly neurological, and so further eliciting the aetiology of NS as what appears to be a self-limiting multi-system neurodegenerative disease may prove not only interesting but also have valuable implications far beyond the narrow geographical confines of this strikingly unusual disorder.

## Supplementary Material

fcac126_Supplementary_Data

## Abbreviations

IDP =: internally displaced persons
MRC =: Medical Research Council
NGO =: non-governmental organization
NS =: nodding syndrome
WHO =: World Health Organization
